# On a Case of the Spontaneous Reproduction of Cowpock

**Published:** 1850-07

**Authors:** 


*On a Case of the Spontaneous Reproduction of Cowpock.* By Dr. Rupprecht.—Dr. Rupprecht reports the case of a girl (æt. 14) who was seized with influenza. She complained of pain in each arm at the spots where, when an infant, she had been vaccinated; and in fact at these localities, vaccine vesicles now became perfectly developed. An elder sister was revaccinated with the lymph hence obtained; beautiful vesicles formed, and ran a normal course.
*Casper's Wochenschrift*, 1849, No. 51.

